# Palmitoylation and regulation of potassium-dependent sodium/calcium exchangers (NCKX)

**DOI:** 10.1042/BSR20241051

**Published:** 2025-01-21

**Authors:** Ran Tao, Alan D. Robertson, William Fuller, Caglar Gök

**Affiliations:** ^1^School of Cardiovascular and Metabolic Health, Sir James Black Building, University of Glasgow, Glasgow, G12 8QQ, U.K; 2 School of Natural Sciences, College of Health and Science, University of Lincoln, Lincoln, LN6 7TS, U.K

**Keywords:** Ca^2+^homeostasis, NCKX, palmitoylation, Solute Carrier (SLC) proteins

## Abstract

Cellular Ca^2+^ homeostasis is critical for normal cell physiology and is regulated by several mechanisms. Two major players in intracellular Ca^2+^ homeostasis in multiple tissues belong to the SLC8 (Na^+^/Ca^2+^ exchangers (NCXs); NCX1-3) and SLC24 (K^+^ dependent Na^+^/Ca^2+^ exchangers (NCKXs); NCKX1-5) families. It has been established that NCXs and NCKX4 are palmitoylated, and that palmitoylation promotes NCX1 inactivation. However, there is still little known about NCKXs’ palmitoylation. We found that (1) NCKX3 and NCKX5, but not NCKX1, are palmitoylated, (2) Cys to Ala mutation at position 467 for NCXK3 and 325 for NCKX5 notably diminished palmitoylation and (3) reduced palmitoylation enhanced NCKX3 activity. Our findings bring novel insights into NCKX1, NCKX3 and NCKX5 palmitoylation and establish palmitoylation as an endogenous regulator of NCKX3 activity, paving the way for investigations evaluating the role of palmitoylation in NCKX3 function in health and disease.

## Introduction

Calcium (Ca^2+^) ions are key regulators of various physiological and biochemical processes (i.e., acting as a second messenger in signal transduction pathways, cell-to-cell communication, neurotransmitter release and cell growth) across multiple tissues. Maintaining intracellular Ca^2+^ [Ca^2+^]_i_ levels at physiological range (~100 nM) [1] and a ~ 10,000-fold concentration gradient of Ca^2+^ between the interior and exterior of the cell are essential for normal cell physiology. This is achieved by several different mechanisms in cells, including the super-family of solute carrier (SLC) proteins, which consists of over 400 proteins and includes two major regulators of Ca^2+^ handling in cells: the SLC8 family of sodium/calcium ion exchangers (NCXs) and the SLC24 family of potassium-dependent sodium/calcium exchangers (NCKXs) [2]. The SLC proteins exhibit distinct expression patterns throughout the body, underscoring the tissue-specific regulation of cellular Ca^2+^. For instance, while NCX1 is expressed in the heart, kidney and brain, NCX2 and NCX3 are predominantly found in the brain and skeletal muscle. In addition to NCXs, a tissue-specific expression pattern is also observed within NCKX family: NCKX2, NCKX3 and NCKX4 are widely expressed throughout brain tissue in both neuronal and non-neuronal cells, whereas NCKX1 and NCKX5 exhibit more restricted tissue distribution (NCKX1 in rod photoreceptors and platelets, and NCKX5 in pigment cells) [3–7].

Interestingly, many of SLC proteins (i.e., SLC1 family: EAAC1, GLT-1 and GLAST [8]; SLC2 family: GLUT1 and GLUT4 [9–11]; SLC6 family: dopamine transporter (DAT) [12]; SLC8 family: NCX1, NCX2 and NCX3 [13–17]) are palmitoylated. Protein palmitoylation is the covalent attachment of 16C palmitate to a cysteine (Cys) residue of the proteins through a thioester bond [18]. Palmitoylation alters various aspects of proteins, including stability, sorting, membrane association, and function in cells [19,20].

Recently the potassium-dependent sodium/calcium exchanger 4 (NCKX4) was reported to be palmitoylated at multiple Cys residues: C118, C130, C419 and C425 [3]. NCKX4 belongs to the SLC24 family, which remains understudied. The SLC24 family comprises five members (NCKX1-5) that co-transport Ca^2+^ and K^+^ ions out of the cell by utilizing the transmembrane Na^+^ gradient [21]. Although NCKX proteins play key roles in various biological processes, such as vision, neuronal function, skin pigmentation and olfaction [4], little is known about their regulatory mechanisms.

Amongst all palmitoylated members of the SLC superfamily, NCX1 palmitoylation and its molecular and cellular consequences have been most studied. NCX1 possesses a single palmitoylation site within its intracellular loop. Palmitoylation of the exchanger at this site is controlled by an amphipathic α-helix adjacent to palmitoylated Cys (C739), and palmitoylation is required for appropriate NCX1 activity and balanced [Ca^2+^]_i_ [22]. Impaired NCX1 palmitoylation is associated with cardiac pathologies [23]. These findings related to the regulation of NCX1 (and therefore intracellular Ca^2+^) by palmitoylation serve as a good paradigm, but there is still a gap in understanding of tissue-specific regulation of cellular Ca^2+^ and the control of this by palmitoylation. Therefore, we explored the palmitoylation of NCKX1, NCKX3 and NCKX5, and demonstrated, for the first time, that [1] NCKX3 and NCKX5, but not NCKX1, are palmitoylated [2], NCKX3, but not NCKX5, is present in the surface membrane and [3] reduced NCKX3 palmitoylation, upon mutating Cys at position 467 (C467) to Alanine (Ala), did not affect its abundance on the surface membrane but altered its activity. Taken together, our findings provide new insights into our understanding of how NCKX proteins control Ca^2+^ homeostasis, which may help explain the distinct mechanisms regulating intracellular Ca^2+^ in different tissue types.

## Results

### NCKX3 and NCKX5 but not NCKX1 are palmitoylated

**Figure 1:**
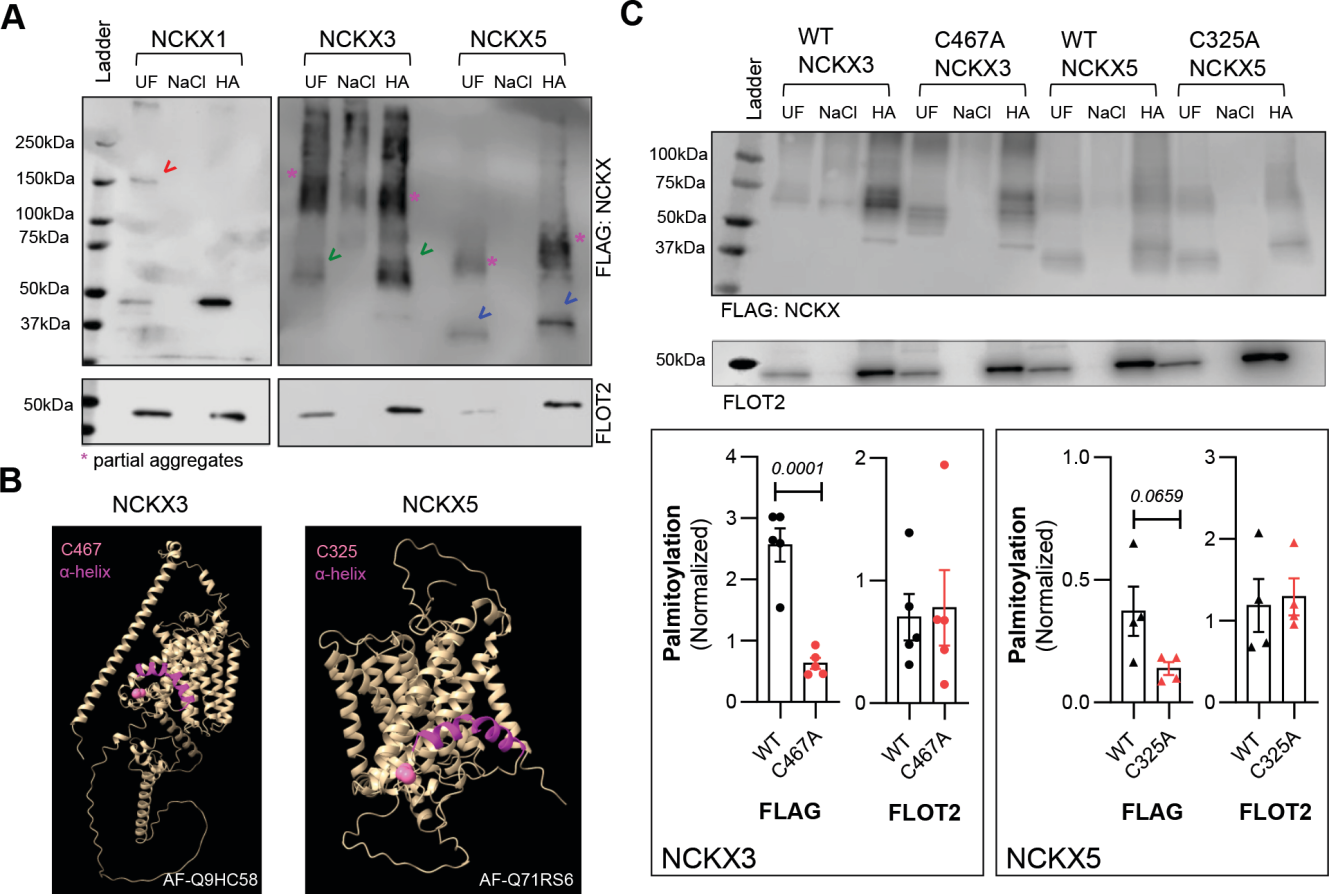
Understanding NCKXs’ palmitoylation. (**A**) NCKX3 and NCKX5, unlike NCKX1, are palmitoylated in HEK293 cells transiently transfected with FLAG-tagged NCKXs (red arrowhead: NCKX1, green arrowhead: NCKX3, blue arrowhead: NCKX5, N:8). (**B**) AlphaFold prediction of NCKX3 (AF-Q9HC58) and NCKX5 (AF-Q71RS6) structure suggested potential (palmitoylatable) Cys residue adjacent to the α-helix (C467 for NCKX3 and C325 for NCKX5) and close to the membrane. Protein structures were built in ChimeraX [24] (**C**) Resin -assisted capture of palmitoylated NCKX3 and NCKX5 from HEK293 cells transiently transfected FLAG-tagged NCKX3 and NCKX5 showed that mutating C467 for NCKX3 and C325 for NCKX5 to Ala significantly reduced palmitoylation (N:5 for NCKX3 and N:4 for NCKX5, unpaired t-test; *P*<0.0001 for WT- vs. C467A- NCKX3 and *P*=0.0659 for WT- vs. C325A- NCKX5). UF: Uunfractionated cell lysate, HA: palmitoylated proteins, NaCl: Nnegative control.

We first tested whether NCKX1, NCKX3 and NCKX5 are palmitoylated. Palmitoylated proteins were purified from HEK293 cells transiently transfected with FLAG-tagged NCKX1, NCKX3 and NCKX5 using resin-assisted capture of acylated proteins (Acyl-RAC). NCKX3 and NCKX5, but not NCKX1, were palmitoylated (Figure 1A). Next, we sought to identify the palmitoylation site(s) for NCKX3 and NCKX5. Unlike NCKX proteins, the structural mechanisms underlying NCX1 palmitoylation are well studied. NCX1 palmitoylation occurs on the Cys residue at position 739 (C739) within the intracellular loop of the exchanger and is governed by the amphipathic α-helix that sits next to C739 [25]. We first examined the predicted structures of NCKX3 (AF-Q9HC58) and NCKX5 (AF-Q71RS6) to identify structural similarities with the NCX1 palmitoylation site. NCKX3 and NCKX5 possess multiple Cys residues; however, we noted a Cys residue (C467 of NCKX3 and C325 of NCKX5) adjacent to an amphipathic helix as in NCX1 (Figure 1B). To explore this further, we introduced Cys to Ala mutation at positions 467 and 325 for NCKX3 and NCKX5, respectively. C467A and C325A mutations reduced the palmitoylation of NCKX3 (by ~4 fold) and NCKX5 (by ~3 fold) (Figure 1C).

**Figure 2:**
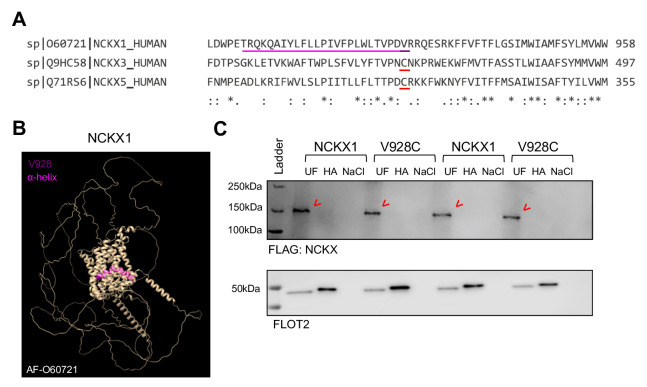
NCKX1 remains unpalmitoylated following V928C mutation. (**A**) Clustal alignment (Clustal Ω) [26]) of NCKX1, NCKX3 and NCKX5 identified Val (underlined in pink) at NCKX1 position 928 in an analogous position to Cys residues (underlined in red) situated adjacent to the α-helix in both NCKX3 and NCKX5 structures. (**B**) Predicted NCKX1 structure (AF-O60721) built in ChimeraX [24]. (**C**) V928C mutation did not induce NCKX1 palmitoylation (red arrowheads, N:4). UF: Uunfractionated cell lysate, HA: palmitoylated proteins, NaCl: Nnegative control.

The Clustal alignment for NCKX1, NCKX3 and NCKX5 (Figure 2A) identified a valine residue (Val) at position 928 adjacent to the α-helix (AF-O60721, Figure 2B) in the analogous position to the palmitoylated cysteines in NCKX3 and NCKX5 [26]. We next tested if the single point mutation from Val to Cys at position 928 in NCKX1 leads to “gain-of-palmitoylation”; however, NCKX1 remained unpalmitoylated in the presence of the V928C mutation (Figure 2C).

### NCKX3 but not NCKX5 is present on surface membrane

**Figure 3:**
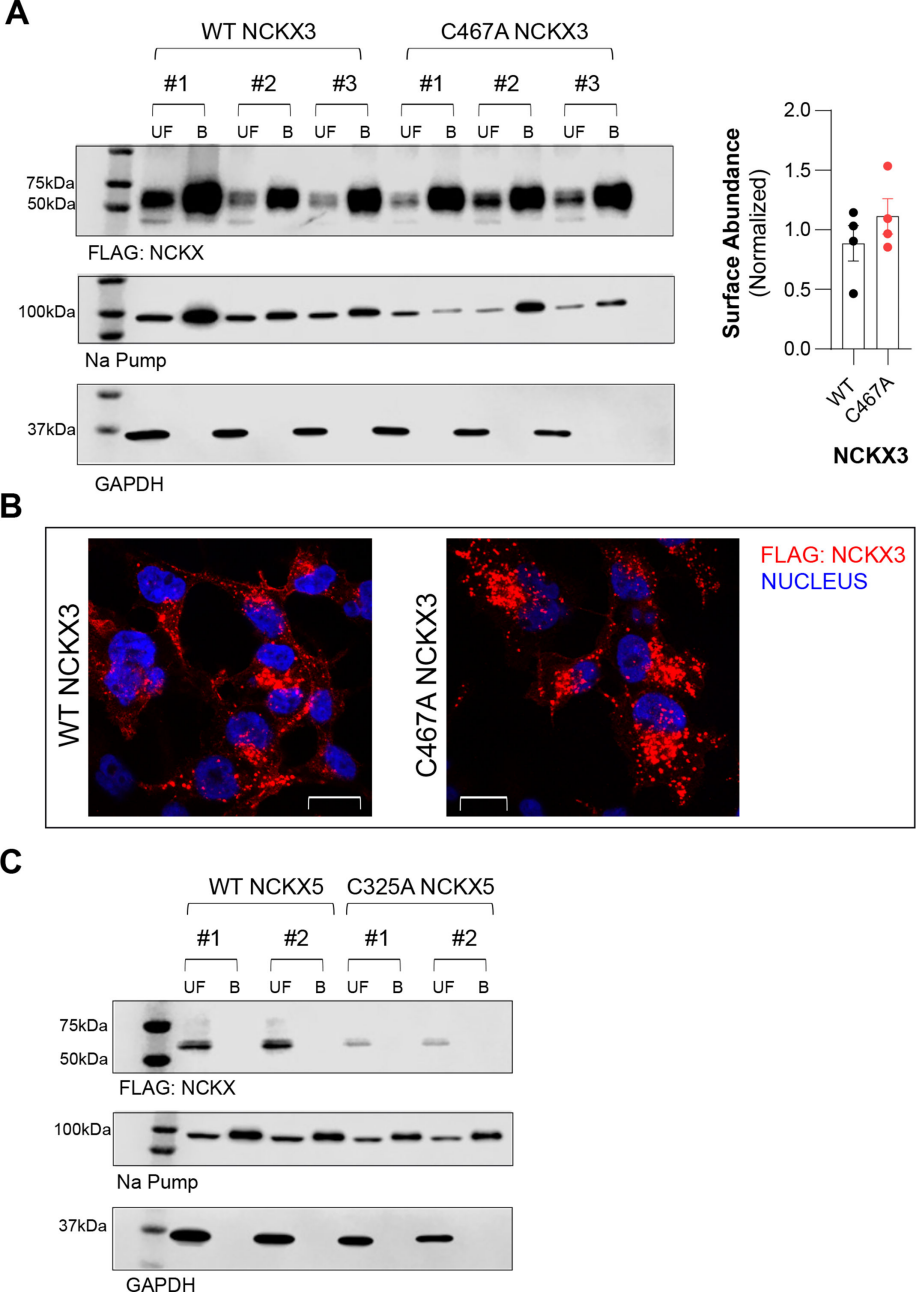
Effect of palmitoylation on subcellular localization. (**A**) Cell surface abundance of WT- and C467A- NCKX3 was similar (unpaired t-test; *P*=0.322 for WT- vs. C467A- NCKX3, N:4). (**B**) WT- (left) and C467A- NCKX3 (right) exhibited similar subcellular localization pattern in Tet- inducible engineered cells: little clusters scattered in the cytoplasm and nucleus (red: FLAG- NCKX3, and blue: Hoechst- nucleus). (**C**) NCKX5 is not present in the plasma membrane. The Na pump is a surface membrane protein and was used to confirm successful purification of surface membrane proteins. Streptavidin- HRP was used to confirm ifwhether surface proteins were successfully probed with biotin and pulled down/captured by streptavidin beads. UF: Uunfractionated cell lysate, UB: unbound fraction, B: Ppurified surface proteins.

Given that palmitoylation controls the subcellular organisation of various proteins, we tested if palmitoylation affects the subcellular localization and surface abundance of NCKX3. Immunofluorescence imaging in tetracycline (Tet-)inducible engineered cells stably expressing wild-type (WT)- or C467A-NCKX3 indicated a similar localization pattern for WT- and mutant NCKX3 (Figure 3A). Besides the plasma membrane, both WT- and C467A-NCKX3 were scattered as small clusters/puncta in the cytoplasm and nucleus. We then assessed the surface abundance of NCKX3 in cells stably expressing tetracycline-inducible WT and C467A NCKX3 cells using a surface biotinylation approach. Surface membrane proteins were labelled with Sulfo-NHS-SS-biotin and captured on the streptavidin beads. No difference in surface abundance between WT- and C467A-NCKX3 was detected (Figure 3B). We did not observe NCKX5 on the surface membrane (Figure 3C).

### Abolishing palmitoylation enhances NCKX3 activity

**Figure 4:**
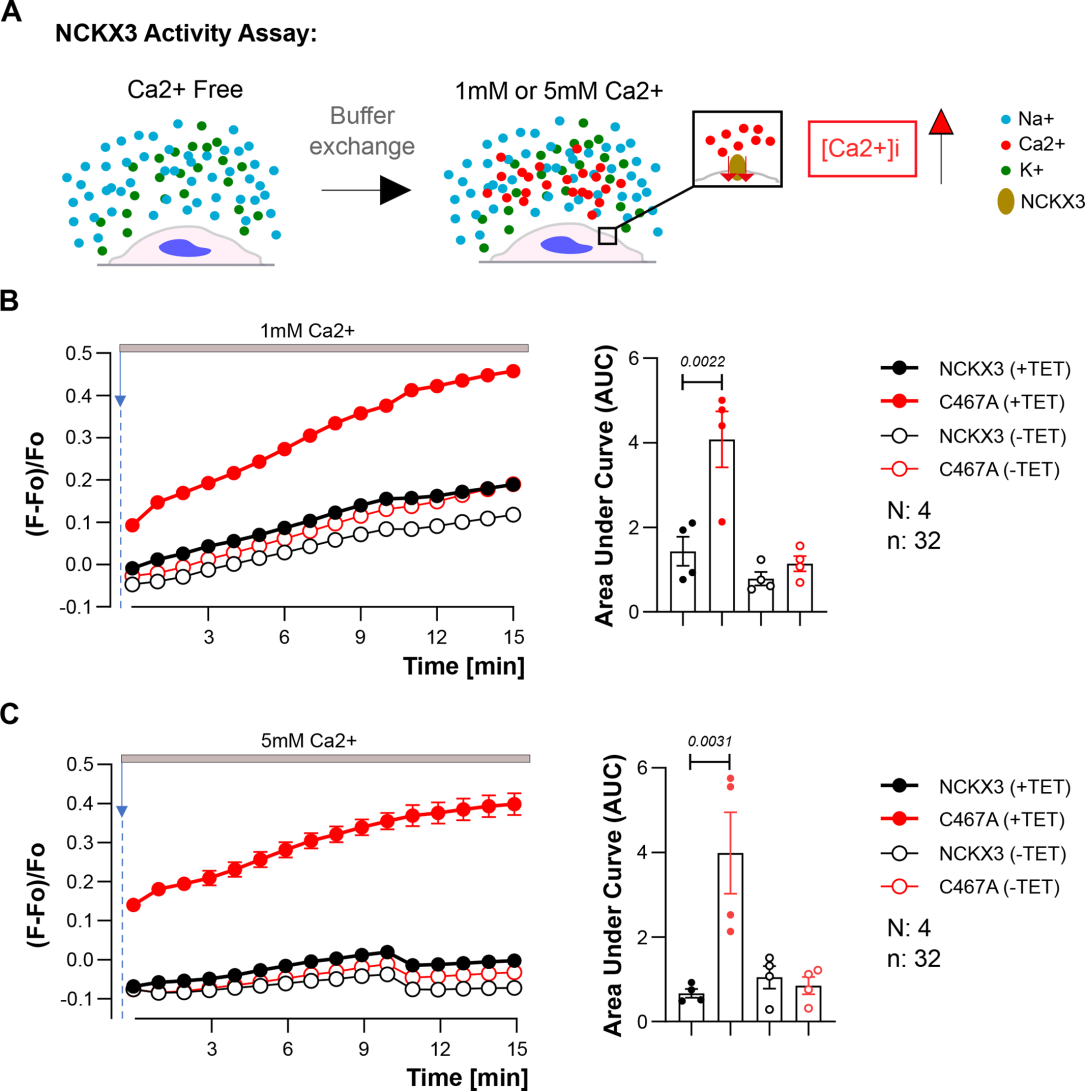
Disrupting NCKX3 palmitoylation increased NCKX3-mediated Ca^2+^ influx. (**A**) Diagram illustrating the NCKX3 activity assay,. (**B, C**) NCKX3 -mediated Ca^2+^ influx was greater in C467A- NCKX3 compared towith WT in Tet- inducible engineered cells upon switching from Ca^2+^-free buffer to 1 mM or 5 mM Ca^2+^-containing buffer (1 mM Ca^2+^: one-way ANOVA with Tukey’s multiple comparison test; *P*=0.0022 for WT (+TET) - vs. C467A (+TET)- NCKX3; 5 mM: one-way ANOVA with Tukey’s multiple comparison test; *P*=0.0031 for WT (+TET) - vs. C467A (+TET)- NCKX3, N:4 and n:32 for each). Ca^2+^ intake was represented/calculated as the area under curve (AUC).

The regulation of ion channel/transporter activity by palmitoylation is well-established for numerous proteins including many Ca^2+^ transport proteins (i.e., NCX1 [15,17], CaV1.2 [27],TRPM7 [28], TRPM8 [29], ORAI1 [30,31], STIM1 [32], SERCA1 [33]). To understand whether NCKX3 activity was regulated by palmitoylation, we measured NCKX-mediated Ca^2+^ influx in cells stably expressing tetracycline inducible WT and C467A NCKX3. Ca^2+^ influx was induced by switching from a Ca^2+^-free extracellular buffer to 1 mM or 5 mM Ca^2+^-containing buffer (Figure 4A). Cellular Ca^2+^-influx was ~3- to 5-fold greater in cells expressing C467A NCKX3 than wild-type cells (Figure 4B and C).

## Discussion

Palmitoylation plays a crucial role in regulating many aspects of protein behaviour (cellular trafficking, stability, function and more) in various tissues. The majority of SLC proteins including the major players of the cellular Ca^2+^ handling (NCX1, NCX2 and NCX3 from SLC8, and NCKX4 from SLC24) are palmitoylated [2]. Palmitoylation of NCXs, particularly NCX1 palmitoylation, and its cellular and physiological consequences are well-studied; however, our knowledge on NCKX palmitoylation was limited to NCKX4. Our work herein set out to investigate the palmitoylation of NCKX1, NCKX3 and NCKX5.

We found that NCKX3 and NCKX5, but not NCKX1, are palmitoylated. The architecture of NCKX proteins contains eleven transmembrane domains (TMs) with an intracellular loop localised between TM5 and TM6 [34]. We identified an amphipathic α-helix in NCKX1, NCKX3 and NCKX5 which, like NCX1, was positioned in this intracellular loop, close to TM6. These α-helices in NCKX3 and NCKX5 (but not NCKX1) are situated adjacent to a Cys residue (C467 for NCKX3, C325 for NCKX5, V928 for NCKX1). Mutating C467 in NCKX3 and C325 in NCKX5 to an Ala-residue significantly diminished the palmitoylation levels of NCKX3 and NCKX5. In contrast, introducing Cys to NCKX1 at position 928 (V928C) did not induce palmitoylation in NCKX1. The features of a substrate protein required for its palmitoylation remain incompletely understood. Clearly, NCKX1 is not recognised by the cellular palmitoylation machinery. We note that the α-helix adjacent to V928 is less amphipathic and more hydrophobic in nature than the equivalent regions of NCKX3 or NCKX5, which we suggest might limit the accessibility of this helical region to the enzyme.

Next, we demonstrated that NCKX3 is predominantly intracellular, with a fraction of the protein present on the surface membrane, as detected by labelling surface proteins with biotin. Furthermore, disrupting NCKX3 palmitoylation did not alter the surface abundance of the exchanger, suggesting that NCKX3 does not need to be palmitoylated to pass through the secretory pathway. In contrast, NCKX5 was not detected on the plasma membrane. Supporting this, previous studies have reported that NCKX5 resides in intracellular membranes with partial colocalization with the trans-Golgi network and mitochondria suggesting that NCKX5 contributes to organellar Ca^2+^ transport [35–37].

Palmitoylation alters the function of numerous proteins [38], and impaired/abnormal palmitoylation is associated with various pathologies [23,39–50]. NCX1 palmitoylation promotes local structural rearrangements within the intracellular loop of the exchanger, which in turn promotes the engagement of Exchanger Inhibitory Peptide (XIP) with its binding site and, therefore, promotes NCX1 inactivation and [Ca^2+^]_i_ balance. Non-palmitoylated NCX1 is less sensitive to XIP and does not properly inactivate [10,12]. In NCKX3 activity assays, we noted that reduced palmitoylation by C467A mutation caused greater Ca^2+^ influx compared with WT-NCKX3, supporting the notion that palmitoylation is an endogenous regulator of NCKX3, and “normal” NCKX3 palmitoylation is critical to protect the cells from Ca^2+^ overload. Given our finding that NCKX3 delivery to the surface membrane is not influenced by palmitoylation, we suggest that the changes in structure or flexibility of NCKX3 induced by palmitoylation modify either the substrate affinities or Vmax of the exchanger, consequently altering cellular Ca^2+^ homeostasis. A paradigm in which palmitoylation of NCKX3 controls intracellular Ca^2+^ concentration has important implications for cellular physiology across multiple tissues where NCKX3 is expressed (e.g., brain, urinary bladder, small intestine [51]).

In conclusion, palmitoylation regulates calcium transport by NCKX3. Our findings suggest that further exploration of the role of palmitoylation in NCKX3 function could shed new light on the relevance of palmitoylation to NCKX3-related pathologies (e.g., abnormal motor function and social behaviour [52], inflammatory bowel diseases [53]).

## Study limitations

We present concise and fundamental observations regarding the palmitoylation of NCKX1, NCKX3 and NCKX5. Mutation of a single Cys to Ala reduced but could not eliminate the palmitoylation of NCKX3 (C467A) and NCKX5 (C325A), implying that there is more than one palmitoylation site in each protein. Therefore, further investigation is needed to comprehensively map the other palmitoylated Cys in the NCKX3 and NCKX5 structures.

We explored neither the enzymatic regulation of NCKX3 and NCKX5 palmitoylation nor the downstream pathway(s) affected by the abnormal exchanger activity due to the impaired palmitoylation. Our activity assays were designed to evaluate palmitoylation-dependent changes in NCKX3 activity by measuring NCKX3-mediated Ca^2+^ influx. We did not address the palmitoylation-dependent changes in the NCKX3 response to K^+^ and Na^+^; therefore, we cannot rule out a potential effect of palmitoylation on K^+^ and/or Na^+^ sensitivity of NCKX3. Hence, it may be necessary to test other ion uptake assays in future experiments.

NCKX5 is not expressed in the plasma membrane but rather on the Golgi and mitochondrial membranes. Assays evaluating the calcium ion transport capacity of NCKX5 on organelle membranes are required to better understand the effects of palmitoylation on NCKX5 function.

All *in vitro* experiments in the current work were performed in HEK293 and the engineered FT293 cells. Although these cells are appropriate models to study palmitoylation of NCKX proteins, additional studies conducted in primary cells and/or animal models are needed to confirm the physiological or pathophysiological relevance of palmitoylation of NCKX proteins in a broader context.

## Future directions

Our findings established that NCKX3 and NCKX5 are palmitoylated, and palmitoylation of NCKX3 alters the exchanger activity. There are, indeed, still more questions to be addressed to see the bigger picture.

### Mapping palmitoylation sites of NCKX3 and NCKX5

Our findings suggest that there are multiple palmitoylation sites within NCKX3 and NCKX5, as the point mutations in NCKX3 (C467A) and NCKX5 (C325A) did not abolish their palmitoylation. Hence, identifying the additional palmitoylation site(s) that exist in NCKX3 and NCKX5 structures would be the next step. This could be achieved by either further mutagenesis (Ala-scanning) studies or mass spectrometry-based proteomics.

### Enzymatic regulation of NCKX3 and NCKX

The palmitoylation and depalmitoylation mechanisms of NCKX3 and NCKX5 remain unknown, and addressing this could help explain whether these proteins are dynamically palmitoylated at the cell surface or if their palmitoylation occurs in the secretory pathways, and which enzymes catalyse their palmitoylation.

### Physiological relevance of NCKX3 and NCKX5 palmitoylation

Validating the findings presented here in different tissues or primary cells from relevant animal models is essential to gain insights into the physiological relevance of palmitoylation of NCKX3 and NCKX5 and its association with the pathologies.

## Methods

### Cells, plasmids and transfection

HEK293 cells and tetracycline (Tet-)inducible FT293 cells stably expressing NCKX3 and NCKX5, engineered using Flp-In T-Rex System (Invitrogen), were used in this study. The HEK293 cells were grown in DMEM supplemented with 10% foetal bovine serum and 1% penicillin and streptomycin in a humidified incubator at 37°C supplemented with 5% CO2. Tet- inducible stable cells were generated by co-transfecting the cells with pcDNA 5 FRT/TO encoding NCKX3 or NCKX5 and pOG44 using GeneJuice (Merck) according to the manufacturer’s instructions, and then selected and maintained in DMEM media containing 100 µg/ml hygromycin.

pDONR221-SLC24A1_STOP was a gift from RESOLUTE Consortium & Giulio Superti-Furga (Addgene plasmid # 161215; http://n2t.net/addgene:161215 ; RRID:Addgene_161215 [54]). Human NCKX3-FlagTag (pcDNA3.1-) was a gift from Jonathan Lytton (Addgene plasmid # 75206; http://n2t.net/addgene:75206 ; RRID:Addgene_75206 [55]). Human NCKX5 (pcDNA3.1+) was a gift from Jonathan Lytton (Addgene plasmid # 75214; http://n2t.net/addgene:75214 ; RRID:Addgene_75214).

Plasmids encoding C-terminally FLAG tagged-NCKX1 and NCKX5 were generated using the InFusion (TakaraBio) cloning strategy. Briefly, pcDNA FRT/TO with a FLAG tag in the multiple cloning site was amplified and ligated to either amplified NCKX1 or NCKX5. The oligonucleotide primers used were GACTACAAGGACGACGATGACAAG and TCCGAGCTCGGTACCAAGC (pcDNA5), GGTACCGAGCTCGGATCCACCATGGGCAAGCTGA and GTCGTCCTTGTAGTCCACGCTCACGGGACAAGAGA (NCKX1), GGTACCGAGCTCGGAATGCAGACAAAAGGGGGCC and GTCGTCCTTGTAGTCACCTCCACAGCCCCTTAT (NCKX5).

Transient transfection of NCKX1, NCKX3 and NCKX5 was achieved using Lipofectamine 2000 (Invitrogen) according to the manufacturer’s instructions.

### Resin-assisted capture of acylated proteins (Acyl-RAC)

Resin-assisted capture of acylated proteins (Acyl-RAC) was performed to purify palmitoylated proteins as described in detail elsewhere [14]. Briefly, free cysteines were first blocked with 1% methyl-methanethiosulfonate (MMTS, Sigma) followed by cleavage of thioester bonds with 250 mM neutral hydroxylamine (HA). The palmitoylation level of NCKX proteins was calculated and presented as the amount of palmitoylated NCKXs purified relative to their abundance in the corresponding unfractionated (UF) cell lysate. Considering the day-to-day variations in palmitoylation stoichiometry, the individual data points were normalised to the experimental mean for that experimental day. FLOT2, in all Acyl-RAC experiments, was probed solely to assess the integrity of the assay, and the enrichment of FLOT2 palmitoylation was neither quantified nor used when calculating the palmitoylation level of proteins of interest.

### Surface biotinylation

Primary amines on surface membrane proteins were biotinylated using 1 mg/mL sulfo-NHS-SS-biotin (APExBIO) in Dulbecco’s PBS (Gibco) and then purified using streptavidin Sepharose as described previously [28]. The intracellular enzyme GAPDH and the surface membrane Na pump were probed as negative and positive controls for the assay and were not considered when calculating the surface abundance of proteins of interest.

### Western blotting

Western blotting employed chemiluminescent detection via horseradish peroxidase (HRP)-based secondary antibody in all experiments described throughout the paper, using the LiCOR imaging system. Band intensities were quantified using densitometry in Image Studio Lite ver 5.2 (LiCOR) software. Antibodies used in this study are FLAG (ProteinTech), GAPDH (Merck), FLOT2 (BD Biosciences), and Na pump (Developmental Studies Hybridoma Bank clone a6f).

### Confocal imaging

Confocal imaging was performed on Tet-inducible engineered cells expressing wild-type or mutated NCKX3, as described in detail elsewhere [14]. Confocal images were captured with Zeiss LSM880 using Airyscan confocal microscopy (63×). The diode (405–430 nm) and argon (458 nm, 488 nm, 514 nm) ion lasers were used for detecting Hoechst and Alexa546. The cells were fixed with 4% PFA in PBS for 10 min and then quenched with 100 mM and 10 mM glycine in PBS incubation (5 min) consecutively. The cell membrane was permeabilised with 0.1% Triton X-100 in PBS, followed by blocking in 3% BSA in PBS at room temperature for 1 h. Samples were incubated overnight at 4°C using FLAG primary antibody (ProteinTech) and then probed with Alexa Fluor 546 (Sigma) secondary antibody at room temperature for 1 h. Hoechst (Invitrogen) was used to label DNA.

### NCKX3 activity assay

Plasmalemmal Ca^2+^ fluxes were measured in tetracycline (Tet)-inducible engineered cells expressing wild-type or mutated NCKX3. Engineered cells (~5 × 10^4^ cells/well) were cultured in 96-well plates coated with poly-L-lysine (PLL, Sigma), and 16–24 h after inducing NCKX3 expression with Tet (1 µg/ml), cells were loaded with Fluo4-Direct (Invitrogen) for 1 h at 37 °C. Fluorescent intensities (Ex: 494 nm and Em:516 nm) were measured using a POLARstar OPTIMA plate-reader (BMG LABTECH). NCKX3 activity was determined as the calcium intake upon triggering Ca^2+^ influx by replacing Ca^2+^-free buffer (140 mM NaCl, 6 mM KCl, 10 mM HEPES, 1 mM EGTA and 5.5 mM glucose, pH:7.4) with Ca^2+^-containing buffer (140 mM NaCl, 6 mM KCl, 10 mM HEPES, 1 mM or 5 mM CaCl_2_ and 5.5 mM Glucose, pH:7.4, Figure 3A). Changes in [Ca^2+^]_i_ were recorded at 1 min intervals upon triggering Ca^2+^ intake by buffer exchange over 15 min. Ca^2+^ intake was calculated and presented as (*F* − *F*_0_)/*F*_0_, where *F*_0_ is the starting fluorescence level in Ca^2+^-free buffer and *F* is the fluorescence level after switching to the buffer with either 1 mM or 5 mM Ca^2+^. The amount of plasmalemmal Ca^2+^ fluxes was calculated as the area under curve (AUC).

### Statistical analysis

All data are presented as mean ± standard error of the mean. Quantitative differences between groups were assessed using unpaired t-tests or one-way ANOVA analysis followed by appropriate post-hoc tests using GraphPad Prism.

## Data Availability

All data generated through this work are available in the manuscript.

## Abbreviations

AUC: Area under curve
Acyl-RAC: acylated proteins
Ala: Alanine
Cys: cysteine
DAT: dopamine transporter
HA: hydroxylamine
HRP: horseradish peroxidase
NCKXs: potassium-dependent sodium/calcium exchangers
NCXs: sodium/calcium ion exchangers
SLC: solute carrier
TMs: transmembrane domains
UF: unfractionated
WT: Wild type
XIP: Exchanger Inhibitory Peptide
